# Bridging the knowledge gap: from microbiome composition to function

**DOI:** 10.15252/msb.20156045

**Published:** 2015-03-11

**Authors:** Jeremiah J Faith

**Affiliations:** Immunology Institute and Institute for Genomics and Multiscale Biology, Icahn School of Medicine at Mount SinaiNew York, NY, USA

## Abstract

Despite the wealth of metagenomic sequencing data, the functions of most bacterial genes from the mammalian microbiota have remained poorly understood. In their recent study (Yaung *et al* 2015), Wang, Gerber, and colleagues present a platform which allows functional mining of bacterial genomes for genes that contribute to fitness *in vivo* and holds great potential for forward engineering microbes with enhanced colonization abilities in the microbiota.

See also: SJ Yaung et al (March 2015)

Next-generation sequencing has enabled a seemingly endless flood of culture-independent characterizations of the human microbiome across different individuals, at different body sites, and under different disease states (Human Microbiome Project C, 2012). Analyses of these metagenomic and 16S rRNA amplicon sequencing data continue to improve our understanding of microbial community composition and dynamics in the human microbiome. However, in our growing enthusiasm of the microbiome and its potential impacts on human health as a prognostic marker, diagnostic tool, or target for disease treatment, it is easy to forget how little we understand of the functional capacities of the microorganisms in this community and how their varied gene content influences their ability to colonize a mammalian host. Every new species detected by 16S rRNA gene amplicon sequencing or metagenomic assembly represents hundreds to thousands of additional genes whose function we do not understand. In their recent study, Yaung *et al* (2015) make important strides to alleviate this functional knowledge gap. They used an approach termed “Temporal Functional Metagenomics sequencing” (TFUMseq) to identify genetic regions that increase microbial fitness in the mammalian intestine (Yaung *et al*, 2015). First, they generated a rich plasmid library of roughly one hundred thousand ∽2.5 kb DNA fragments covering the genome of *Bacteroides thetaiotaomicron*, a common member of the healthy gut microbiota. The plasmid libraries, designed with a strong constitutive promoter to express the cloned genome fragment, were transformed into *Escherichia coli* K-12. To determine if constitutively expressed genetic loci from *B. thetaiotaomicron* modulate the ability of *E. coli* to colonize the mammalian intestine, Yaung *et al* inoculated germ-free animals with the *E. coli* harboring the plasmid library and tracked the abundance of *B. thetaiotaomicron* regions over time by sequencing DNA samples from fecal pellets taken for up to 28 days post-inoculation. Those regions overrepresented over time revealed genes, such as carbohydrate utilization loci, that provide a fitness advantage to the *E. coli* that harbor them.

Similar functional metagenomic approaches have been used *in vitro*, for instance for identifying microbial genetic regions that confer antibiotic resistance (Dantas *et al*, 2013). Moreover, a complementary approach published by Goodman *et al* (2009) used a rich library of insertion sequences in *B. thetaiotaomicron* to probe for genetic regions that modulate colonization by identifying those insertion sequences (and the corresponding genes disrupted by the insertion sequence) that are depleted from the insertion sequence library. An advantage of this method, termed insertion sequencing (INSeq), is that it probes gene function in the context of an organism's native genome rather than by transplanting genes into a foreign host organism, with perhaps markedly different functional potential. These INSeq libraries can provide information about the depletion of tens to hundreds of genes, whereas overexpression-based functional metagenomics methods like TFUMseq might be more biased toward identifying a smaller number of genetic regions that improve fitness. This is due to the fact that large increases in fitness can quickly bottleneck a library into a few dominant loci that outcompete the remainder of the library and limit the breadth of information gathered from any individual experiment. Importantly, Goodman *et al* found numerous genetic regions that were important in the context of a diverse community of microbes but not in the context of mono-colonization. Moving forward, it is essential to apply TFUMseq to microbial communities of increasing complexity.

The framework presented by Yaung *et al* for understanding the functional capacity of gut microbes drives to the heart of the most fundamental requirement of any organism in the microbiome, namely its ability to stably colonize a host in the context of the rich genetic diversity of competing organisms and host factors. Notably, the microbiota in an adult human gut is stable, with the majority of organisms likely remaining there for decades (Faith *et al*, 2013), perhaps by utilizing a variety of recently reported molecular mechanisms that foster stability in both the healthy and inflamed gut (Lee *et al*, 2013; Cullen *et al*, 2015). Therefore, identifying the genetic elements that confer fitness advantages during colonization is essential for understanding the final assembly of each individual's microbiota. Advantages of TFUMseq compared to existing methodologies include its simplicity and, more importantly, its potential to be applied to forward engineer bacterial strains with enhanced colonization abilities in defined contexts, such as specific dietary interventions. Functional metagenomic libraries can be transformed into microbes with established genetic tools, drastically reducing the complexity of experimental implementation in the context of gut microbes, most of which lack tools for genetic manipulation. Libraries from the same genetic pool can be tested in several different hosts to understand the influence of host organism genome on the functional contribution of each element in a library. The libraries themselves are not necessarily limited to the genetic material of individual organisms, but they could also represent a pool of the genetic content of numerous organisms, allowing the parallel analysis of multiple genomes worth of genetic potential. These advantages of TFUMSeq will hopefully lead to its wide application in a diversity of contexts and will help bridge the gap between our knowledge of microbiome composition and function.

**Figure 1:**
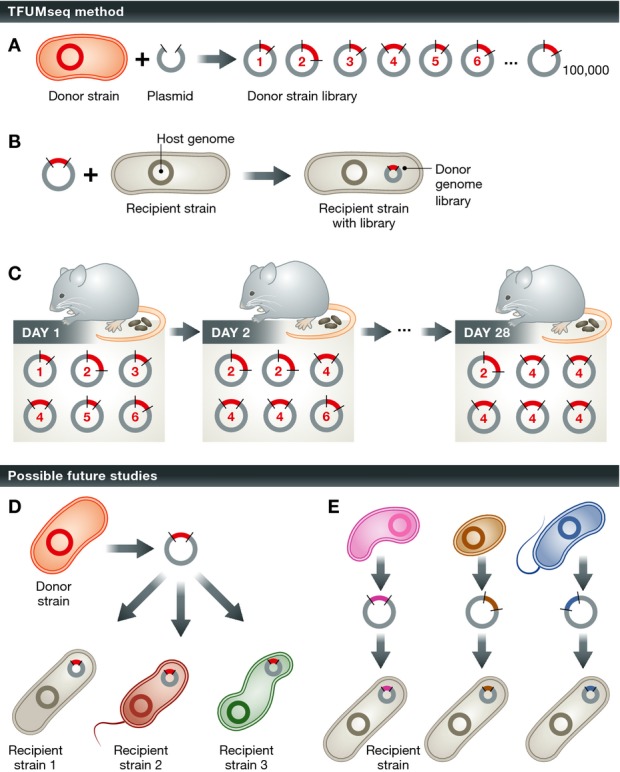
Overview of temporal functional metagenomics sequencing (TFUMseq). A plasmid library of approximately one hundred thousand ∽2.5 kb DNA fragments covering the genome of *B. thetaiotaomicron* was generated (A) and transformed into *E. coli* (B). The abundance of *B. thetaiotaomicron* regions over time (up to 28 days post-inoculation) was tracked by sequencing DNA samples from fecal pellets (C). The regions that are overrepresented over time (i.e. #4) indicate genes that provide a fitness advantage. (D, E) Future applications of TFUMseq. (D) Libraries from the same donor strain can be tested in several different recipient strains. (E) Libraries representing a pool of the genetic content of numerous organisms can be used, allowing the parallel analysis of multiple genomes.
